# The mechanism of oxidative stress in asthenozoospermia and antioxidant strategies: a review

**DOI:** 10.3389/fendo.2025.1670762

**Published:** 2025-10-01

**Authors:** Linfeng Mo, Hongmei Wu, Mingxue Zhang, Peng Zhang, Wei Peng, Yonghua He, Feng Gao

**Affiliations:** ^1^ Department of Health Management, Guangzhou Huashang Vocational College, Guangzhou, China; ^2^ Department of Medical Records, Occupational Diseases Hospital of Shandong First Medical University/Shandong Province Hospital of Occupational Diseases, Jinan, China; ^3^ Department of Medical Insurance and Price, Medical Center Hospital of Qionglai City, Chengdu, China; ^4^ Department of Epidemiology and Health Statistics, Guilin Medical University, Guilin, China

**Keywords:** oxidative stress, asthenozoospermia, sperm motility, antioxidants, fertility

## Abstract

Asthenozoospermia, a leading cause of male infertility, is closely associated with oxidative stress (OS), which reflects an imbalance between reactive oxygen species (ROS) production and antioxidant capacity. ROS originate from both endogenous sources (e.g., inflammation and infection) and exogenous exposures (e.g., lifestyle and environmental pollutants). At physiological levels, ROS participate in key processes such as sperm proliferation, maturation, capacitation, acrosome reaction, and fertilization. However, excessive ROS become detrimental, damaging sperm membrane lipids, DNA integrity, and mitochondrial function, ultimately leading to reduced sperm motility and impaired fertility. A systematic understanding of the sources and mechanisms of ROS in asthenozoospermia is essential for developing targeted interventions. This review highlights the comprehensive integration of multiple ROS sources and their multi-level damaging effects, with a particular focus on mitochondrial dysfunction as a central mechanism in OS-induced sperm damage. Furthermore, we discuss the potential of antioxidant-based strategies and propose future directions for targeted therapies. This work aims to provide new insights into the treatment of asthenozoospermia and facilitate a shift from empirical management to mechanism-targeted therapies in clinical practice.

## 1 Introduction

Infertility is a common reproductive health issue, with male factors accounting for approximately half of all cases of infertility (1). Asthenozoospermia is one of the primary manifestations, accounting for approximately 18% in clinical settings (2–4). The disease is primarily characterized by a forward progression rate of less than 32%, with its core issue being impaired sperm motility. This defect hinders the sperm’s ability to reach and penetrate the egg, resulting in fertilization failure. Sperm motility is highly dependent on energy supplied by mitochondria (5). At the same time, mitochondria play a crucial role in reactive oxygen species (ROS) signaling, calcium homeostasis, steroid hormone biosynthesis, and apoptosis (6, 7). Under physiological conditions, mitochondria generate certain levels of reactive oxygen species (ROS), including superoxide anion (O_2_^·-^), hydrogen peroxide (H_2_O_2_), and hydroxyl radical (·OH) (8). These ROS play essential physiological roles in mediating sperm capacitation, hyperactivation, acrosome reaction, and fusion with the oocyte (9). However, when ROS production exceeds the body’s antioxidant capacity, oxidative stress (OS) is induced, which damages both nuclear and mitochondrial DNA in sperm, ultimately leading to male infertility (10).

OS refers to a pathological state characterized by an imbalance between oxidative and antioxidant systems (11). Under physiological conditions, both enzymatic and non-enzymatic antioxidant substances present in seminal plasma effectively scavenge ROS and maintain redox homeostasis (6, 12, 13). However, this balance is disrupted when ROS production becomes excessive or when antioxidant defense mechanisms are compromised (e.g., decreased antioxidant enzyme activity), leading to the onset of OS (14, 15). Studies have shown that OS is closely associated with various male reproductive disorders and represents a key mechanism underlying sperm dysfunction (16–19). Elevated ROS levels can damage sperm membrane lipids, proteins, and nucleic acids, causing DNA fragmentation and errors in transcription and translation, ultimately impairing sperm motility and fertilizing capacity (20, 21).

Existing research has recognized the important role of OS in asthenozoospermia. However, a comprehensive integration of the diverse sources of ROS is still lacking. Furthermore, there is no systematic elucidation of the multi-level damaging mechanisms. This is especially true for understanding the central role of mitochondrial dysfunction within the entire regulatory network. Moreover, the translation of molecular mechanisms into clinical intervention strategies remains insufficient. Based on a systematic review of existing literature, this article comprehensively summarizes how ROS is generated from both endogenous and exogenous sources. The article also provides an in-depth analysis of the pathophysiological relationship between OS and asthenozoospermia. Finally, it explores the underlying molecular mechanisms and potential therapeutic targets. It aims to offer a theoretical basis and novel perspectives for the precise diagnosis and targeted treatment of asthenozoospermia.

## 2 Sources of ROS

### 2.1 Endogenous sources

**Figure 1:**
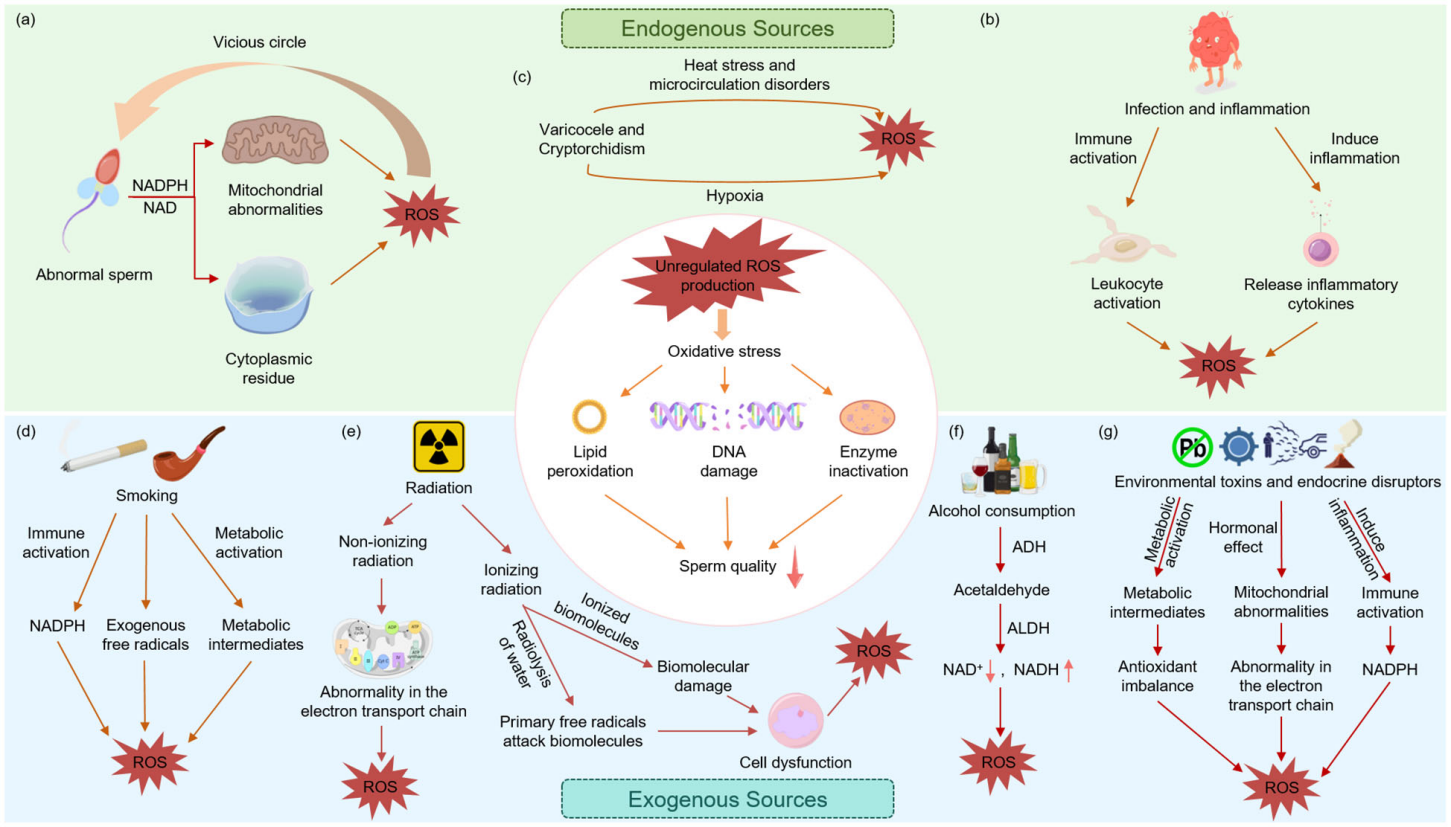
Generation of excess ROS by endogenous and exogenous sources. Diagram illustrating sources and effects of ROS on sperm quality. Endogenous sources include **(a)** abnormal sperm, **(b)** infection and inflammation, and **(c)** varicocele and cryptorchidism. Exogenous sources comprise **(d)** smoking, **(e)** radiation, **(f)** alcohol consumption, and **(g)** environmental toxins and endocrine disruptors. All pathways lead to ROS overproduction, resulting in lipid peroxidation, DNA damage, enzyme inactivation, and impaired sperm quality.

Sperm generate ROS through two primary pathways: one involves the reduced nicotinamide adenine dinucleotide phosphate (NADPH) oxidase located on the plasma membrane, and the other occurs via nicotinamide adenine dinucleotide (NAD)-dependent redox reactions (22). During spermatogenesis, defective cytoplasmic extrusion leads to the retention of excess residual cytoplasm (ERCs), resulting in morphologically abnormal sperm. These residual cytoplasmic droplets are rich in metabolic enzymes such as glucose-6-phosphate dehydrogenase (G6PD) and NADPH oxidase, which can persistently activate ROS-producing pathways and significantly elevate ROS levels (**Figure 1a**) (23).

Inflammation and infection represent another major endogenous source of ROS. Activated leukocytes generate large quantities of ROS through the “respiratory burst” (24), an immune defense mechanism that can increase ROS production by orders of magnitude above basal levels. This process is further amplified via the pentose phosphate pathway, which enhances NADPH generation and exacerbates OS (**Figure 1b**) (25). Both bacterial prostatitis (26, 27) and other pathogenic infections—such as those caused by herpes simplex virus (HSV) (28), human immunodeficiency virus (HIV) (29), hepatitis viruses (30, 31), and Mycobacterium tuberculosis (32) —can trigger leukocyte-dependent ROS elevation. Chronic non-bacterial (non-infectious) prostatitis is also strongly associated with increased OS levels in semen (33). Furthermore, under stimulation by inflammatory cytokines (e.g., IL-6, IL-8, TNF-α), both somatic and spermatogenic cells within the testes can contribute to ROS overproduction and reduced antioxidant capacity, leading to oxidative damage (34).

Certain male reproductive disorders are also closely associated with elevated ROS levels. Varicocele, characterized by increased testicular temperature and local hypoxia, can induce oxidative stress and impair testicular function (**Figure 1c**) (35, 36). Studies have shown significantly elevated ROS and lipid peroxidation levels in the semen of affected patients (35, 37), which correlate positively with sperm DNA fragmentation rates (38). Even after surgical correction via orchiopexy, patients with cryptorchidism continue to exhibit increased ROS production and aggravated DNA damage (39). Testicular torsion, on the other hand, triggers testicular damage through ischemia-reperfusion injury, leading to leukocyte infiltration and a burst of free radicals that ultimately impair spermatogenesis (1, 37, 40).

Endocrine and metabolic disorders also contribute to ROS-mediated spermatogenic damage. Diabetes mellitus is associated with increased oxidative DNA damage in sperm (39). Systemic conditions such as chronic kidney disease and hemoglobinopathies (e.g., β-thalassemia) can similarly induce oxidative sperm damage due to reduced antioxidant capacity (41–43). These mechanisms collectively lead to impaired sperm membrane integrity, DNA fragmentation, and functional abnormalities, and may even affect the recovery of reproductive function following vasectomy reversal (44–46).

### 2.2 Exogenous sources

Exogenous sources of ROS primarily include physical, chemical, and lifestyle factors. Unhealthy lifestyles can trigger excessive ROS production through immune cell activation, depletion of antioxidant reserves, and promotion of pro-oxidative reactions. Smoking increases leukocyte counts in semen by 48% and elevates ROS levels by 107%, while reducing total antioxidant capacity, ultimately leading to germ cell apoptosis and DNA damage (22, 47). Heavy metals in tobacco (e.g., cadmium and lead) further augment ROS levels and impair sperm motility (22). Alcohol consumption, through its metabolite acetaldehyde, also promotes ROS generation and compromises sperm function (**Figures 1d, f**) (48).

The testes, which rely on superficial thermoregulation, are particularly sensitive to non-ionizing radiation. Studies indicate that exposure to non-ionizing radiation such as mobile phone emissions can elevate scrotal temperature, reduce antioxidant enzyme activity, disrupt mitochondrial function, and promote ROS generation in seminal plasma, ultimately leading to DNA damage and impaired sperm parameters (40, 49). Radiofrequency electromagnetic fields (RF-EMF), through both thermal and non-thermal effects, interfere with the electron transport chain and cellular membranes, induce oxidative stress and DNA fragmentation, and impair steroidogenic function (**Figure 1e**) (22, 50, 51).

Chemical agents represent another significant exogenous source of ROS. Endocrine-disrupting chemicals (e.g., phthalates) from industrial products and plastics, as well as heavy metals (e.g., lead, cadmium, and mercury), can enter the human body through various routes. These compounds induce excessive ROS production by depleting antioxidants, activating enzymatic ROS-generating systems (e.g., NADPH oxidase), and triggering mitochondrial dysfunction, ultimately impairing spermatogenesis and sperm quality (**Figure 1g**) (32, 47, 52).

Despite their diverse origins, both endogenous and exogenous factors converge on a common pathogenic pathway: by inducing mitochondrial dysfunction, activating enzymatic ROS-producing systems, or impairing antioxidant defenses, they lead to excessive ROS accumulation. This subsequently damages sperm membrane integrity, DNA stability, and motility, ultimately resulting in male infertility.

## 3 OS: The dual role of ROS and multi-targeted damage mechanisms in asthenozoospermia

**Figure 2:**
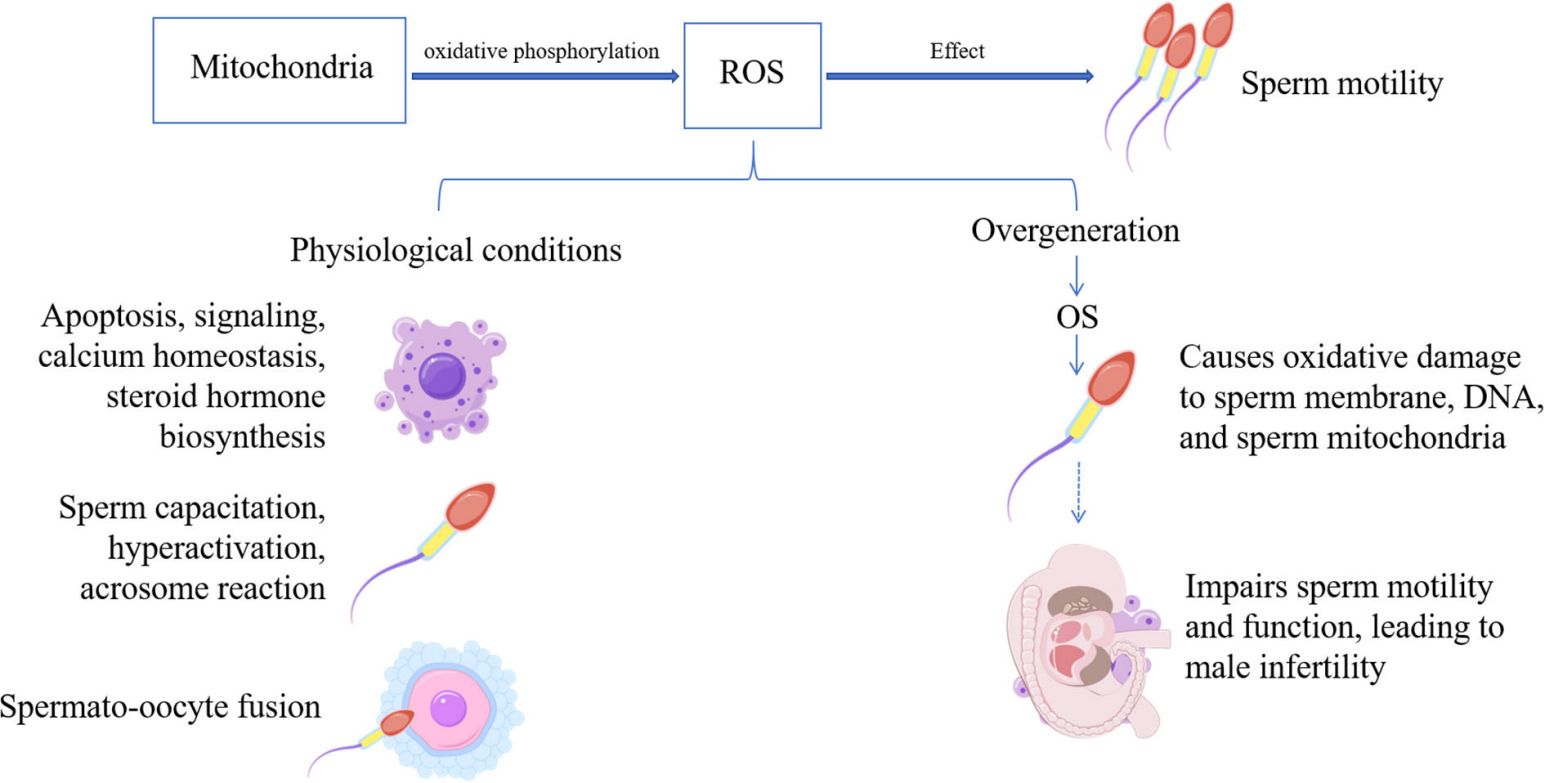
Physiological effects of ROS and the hazards of excesses.

The concept of OS was first introduced by Helmut Sies in 1985 (53). Subsequent research has progressively elucidated its central role in impairing male reproductive function (54). As mitochondria-rich cells, sperm require physiological levels of ROS for successful fertilization (55). However, excessive ROS induces multi-target damage, including: triggering lipid peroxidation, which disrupts membrane fluidity and structural integrity; causing nuclear and mitochondrial DNA fragmentation; impairing plasma membrane function; and leading to mitochondrial dysfunction with compromised ATP synthesis (**Figure 2**) (56). These alterations are particularly prominent in patients with asthenozoospermia (57). Together, these mechanisms contribute to reduced sperm motility, functional defects, and loss of fertilizing potential, forming a critical molecular basis of male infertility. Notably, excessively high antioxidant concentrations can also be detrimental by inducing reductive stress, which is equally damaging as OS (58). Therefore, the key to managing OS lies in the precise regulation of both ROS and antioxidant levels.

### 3.1 Oxidative damage to the sperm membrane

The sperm membrane is rich in polyunsaturated fatty acids (PUFAs), whose double bonds weaken adjacent C–H bonds and increase susceptibility to OS. Membrane-embedded proteins involved in signal transduction, ion channels, and cell adhesion further enhance its vulnerability to oxidative attack (59). ROS originate from multiple sources, including mitochondrial electron leakage in sperm, dysfunction of endogenous antioxidant enzymes, and exogenous factors such as smoking or radiation (60). These reactive species induce lipid peroxidation, disrupting membrane architecture, fluidity, integrity, and protein function. This leads to ion dysregulation, interrupted signaling, and activation of apoptosis, ultimately resulting in loss of motility and reduced fertilizing capacity (61, 62). Moreover, OS-induced lipid peroxidation compromises mitochondrial membrane integrity, reduces membrane potential, inhibits ATP synthesis, and alters cellular energy metabolism, thereby creating a vicious cycle of further ROS accumulation (63). Consequently, maintaining an effective antioxidant defense is essential for sperm health in the context of environmental and lifestyle challenges. Interventions such as a balanced diet, regular exercise, and avoidance of harmful exposures may help mitigate the negative impact of OS on sperm function (64).

### 3.2 Oxidative damage to DNA

The integrity of sperm DNA is essential for successful fertilization and healthy embryonic development. Alterations in DNA structure can directly affect gene expression and protein function, thereby compromising fertilization potential (10). At physiological concentrations, ROS act as crucial signaling molecules in fertilization-related processes. Their small molecular size, rapid generation, and short half-life make them well-suited as intracellular messengers (65). ROS modulate sperm capacitation, motility, and egg-binding ability through activation of the cAMP/PKA pathway and facilitate sperm-egg fusion (22, 66). However, excessive ROS disrupt redox homeostasis and induce DNA base modifications and strand breaks, representing a key mechanism of sperm DNA fragmentation (67). OS can disturb the antioxidant enzyme balance in both seminal plasma and sperm, damaging DNA structure and function (68), undermining genetic stability, and reducing fertilization success (69, 70). OS may also interfere with histone-to-protamine exchange, leading to abnormal chromatin condensation and impaired sperm function (61, 71–73). Furthermore, lipid peroxidation (LPO) products, such as reactive aldehydes, can exacerbate nuclear DNA damage and membrane dysfunction, ultimately hindering the fertilization process (62, 74).

Sperm motility is highly dependent on mitochondrial ATP supply, and the integrity of mitochondrial DNA (mtDNA) is critical for energy metabolism. Located within the mitochondrial matrix, each mitochondrion contains one or multiple copies of mtDNA (75–77), which is maternally inherited (78). Compared to nuclear DNA, mtDNA lacks histone protection, has limited repair capacity, and is more prone to mutation, with a mutation rate approximately 10–20 times higher than that of nuclear DNA (76, 79). Elevated ROS levels can directly damage mtDNA, causing strand breaks and mutations that impair its transcription and replication, ultimately disrupting the synthesis of oxidative phosphorylation proteins and compromising energy homeostasis (80). When the proportion of mutated mtDNA exceeds a critical threshold, cellular energy output declines, leading to sperm dysfunction and related clinical manifestations. Multiple studies have identified mtDNA mutations in infertile men affecting genes involved in the oxidative phosphorylation pathway (81). These mutations result in insufficient ATP synthesis, markedly reduced sperm motility, and diminished fertility (82).

OS-induced DNA damage plays a central role in the pathogenesis of asthenozoospermia, extending beyond mere genetic disruption to multidimensional mechanisms that collectively contribute to sperm motility failure. These interconnected processes lead to deficiencies in structural proteins essential for flagellar movement, disruption of energy supply, and dysregulation of signaling pathways, thereby elucidating the molecular basis of impaired motility in asthenozoospermia. Strategies aimed at protecting and repairing oxidative DNA damage may offer critical therapeutic targets for improving sperm motility.

### 3.3 Oxidative damage to sperm mitochondrial function

Sperm motility is highly dependent on ATP generated via mitochondrial oxidative phosphorylation, and impairment of this process directly leads to reduced sperm vitality and male infertility. Factors such as mitochondrial Ca^2+^ overload (83) or deficiency of cytochrome c (84) can cause electron leakage from the electron transport chain, resulting in excessive ROS production and sustained OS (18, 85). Additionally, compromised mitochondrial membrane integrity and abnormalities in the fibrous sheath can adversely affect sperm function and fertilization (86, 87). Elevated ROS levels can oxidatively modify key respiratory enzymes, such as succinate dehydrogenase and cytochrome c oxidase, impairing their catalytic activity and electron transfer function (88). Furthermore, OS promotes the release of pro-apoptotic factors like cytochrome c from mitochondria, activating caspase-dependent apoptotic pathways and triggering programmed sperm death (89). Studies indicate that alterations in mitochondrial respiratory enzyme activity are significantly associated with idiopathic asthenozoospermia, offering new insights into its molecular mechanisms and identifying potential therapeutic targets (90).

Mitophagy is a critical mechanism for clearing damaged mitochondria and maintaining cellular homeostasis. This process is initiated by specific receptor pathways in response to signals such as mitochondrial depolarization, ROS, and hypoxia. While OS can activate autophagy, it may also disrupt mitochondrial protein homeostasis and impair the cell’s ability to clear abnormal proteins (91), leading to the accumulation of damaged mitochondria and persistent ROS production, which further deteriorates the intracellular environment (92). Moreover, OS can exacerbate mitochondrial dysfunction and cellular stress responses by activating transcription factor pathways such as NF-κB and p53 (93). A comprehensive evaluation of sperm mitochondrial function typically includes indicators such as mitochondrial membrane potential, respiratory chain activity, and calcium ion homeostasis. These parameters collectively reflect mitochondrial physiological status and possess predictive value for sperm motility (94, 95). In-depth research on the regulatory mechanisms of mitochondria in sperm will not only help elucidate the etiology of male infertility but also provide new insights for optimizing assisted reproductive technologies (ART).

In summary, OS contributes significantly to the pathogenesis of asthenozoospermia by impairing mitochondrial function and disrupting mitophagic homeostasis. On the one hand, ROS induce loss of mitochondrial membrane potential, inhibit ATP synthesis, and cause energy failure, further amplifying ROS accumulation. Concurrently, OS dysregulates mitophagy—either by excessive activation leading to degradation of healthy mitochondria, or by impairing clearance mechanisms resulting in accumulation of damaged organelles—thereby exacerbating oxidative damage and apoptotic signaling. These processes form a self-amplifying vicious cycle that ultimately leads to reduced sperm motility, functional defects, and loss of fertilizing capacity. Restoring mitochondrial function and mitophagic balance may therefore represent a promising therapeutic strategy for asthenozoospermia.

## 4 Antioxidant strategies and clinical applications

Antioxidants are categorized into two major groups based on their activity and chemical structure: enzymatic and non-enzymatic antioxidants (96). Enzymatic antioxidants rely on trace elements such as zinc, iron, magnesium, and copper as cofactors to catalyze the conversion of ROS into hydrogen peroxide and subsequently reduce it to water (97). This process protects sperm from lipid peroxidation and OS, thereby helping maintain sperm motility and vitality (98). Zinc is not only involved in germ cell development and the synthesis of luteinizing hormone, follicle-stimulating hormone, and testosterone (99), but also serves as an essential component of various antioxidant enzymes, playing a key role in mitigating oxidative damage and improving sperm quality (100, 101). Selenium is another crucial trace nutrient that influences spermatogenesis and testosterone synthesis. Twenty-five selenoproteins have been identified in humans and animals, many of which are vital for maintaining sperm structural and functional integrity (102).

Non-enzymatic antioxidants, such as vitamin C, vitamin E, and melatonin, mitigate oxidative damage by directly neutralizing free radicals and interrupting chain reactions (103). Studies have shown that sperm with elevated ROS levels often exhibit reduced vitamin C content (102). As the primary water-soluble antioxidant in extracellular fluids, vitamin C not only suppresses ROS generation but also protects sperm DNA integrity by regenerating oxidized tocopherol and scavenging hydroxyl radicals (104). Vitamin E, primarily composed of tocopherols and tocotrienols, is abundant in wheat germ, avocados, and vegetable oils (102). Its phenolic hydroxyl group reacts directly with lipid peroxyl radicals, thereby blocking the progression of membrane lipid degradation (105). Furthermore, the combination of vitamins C and E demonstrates a synergistic protective effect, more effectively defending against peroxidative damage and DNA strand breaks (102).

Numerous clinical trials have demonstrated the beneficial effects of antioxidant supplementation in alleviating OS in patients with asthenozoospermia. Compounds such as L-carnitine, folic acid, and coenzyme Q10 (CoQ10) have been shown to significantly reduce sperm DNA fragmentation while improving sperm concentration and total motility (106–109). An Italian study reported an average increase of approximately 20% in sperm motility following antioxidant intervention (110). Other studies have also indicated that antioxidant supplementation improves sperm count, morphology, and OS levels, accompanied by higher fertilization rates and increased proportions of high-quality embryos (111). Plant-derived antioxidants, such as green tea catechins, have also shown potential in enhancing sperm quality (100, 112). Additionally, astaxanthin, N-acetylcysteine, vitamin E, β-carotene, and unsaturated fatty acids—whether used individually or in combination—have been proven effective in reducing ROS levels (113–115). The majority of published studies support the role of antioxidant therapy in improving sperm parameters and pregnancy outcomes (116–118).

However, notable inconsistencies and even contradictory conclusions exist among different clinical trials. For instance, studies by Alahmar, Cheng, and Sadaghiani et al. reported varying degrees of improvement in sperm function following antioxidant intervention (119–123), which may be attributed to heterogeneity in patient baseline OS status, extent of mitochondrial dysfunction, intervention dosage, and study design. Particularly noteworthy is a randomized controlled trial indicating that combined vitamin C and E supplementation did not significantly improve sperm function (105), underscoring the current lack of consensus. **Table 1** summarizes commonly used antioxidants and their recommended dosages in recent clinical trials targeting asthenozoospermia.

**Table 1:** Antioxidants commonly used in clinical trials for the treatment of asthenozoospermia.

Classifications	Antioxidants	Types of research design	Sample size	Consumption and length of consumption	Main research results (quantitative indicators)	Clinical relevance/impact	Major limitations of the study	References
Trace elements	Zinc	Clinical trial	120	Two zinc sulfate capsules (220 mg each) every day, 3 months	The sperm volume, sperm count, progressive sperm motility and normal sperm form increased by 30.6%, 48.9%, 85.7% and 57.1%	Significantly improve semen parameters	1. Potential bias due to the single-blind study design 2. Absence of a placebo control group 3. Geographical limitations of single-center studies	Alsalman et al. (2018) (133)
	Selenium	Clinical trial	115	200 μg/day, 6 months	The sperm concentration, progressive motility and total motility increased by 36.3%, 76.0%, 28.0%, sperm DNA fragmentation decreased by19.3%	Significantly improve sperm quality and DNA integrity	1. Single-blind or double-blind design was not strictly implemented 2. Absence of a placebo control group 3. Geographical limitations of single-center studies	Alahmar et al. (2023) (134)
Vitamins	Vitamin E	Randomized controlled trial	106	Vitamin E 100 mg/tid, 3 months	The progressive sperm motility increased by 16.4%, the natural pregnancy rate increased by 63.6%	Significantly improve semen parameters and increase pregnancy rates	Multicenter but with uneven sample distribution	Chen et al. (2012) (135)
	Vitamin D3	Randomized controlled trial	86	4000 IU/d, 3 months	The sperm total motility and progressive sperm increased by15.1%, 26.1%	Improve the motility of sperm	Geographical limitations of single-center studies	Maghsoumi-Norouzabad et al. (2021) (136)
Coenzymes	CoQ10	Randomized controlled trial	65	200 mg/day or 400 mg/day, 3 months	The sperm concentration, progressive motility, total motility increased by 62.6%, 83.5%, 48.4%	Significantly improve sperm motility	1. Absence of a placebo control group 2. Geographical limitations of single-center studies 3. Potential bias due to the single-blind study design	Alahmar et al. (2019) (119)
	CoQ10	Clinical trial	85	300 mg/day, 3 months	The sperm progressive motility and total motility increased by 20.8%, 23.4%	Improve sperm function and oxidative balance	1. Absence of a placebo control group 2. Geographical limitations of single-center studies	Alahmar (2022) (120)
Amino acids	L-carnitine	Randomized controlled trial	143	15 g/bag, orally one bag at a time, twice a day, 3 months	The sperm concentration, progressive motility and normal sperm form increased by 30.6%, 49.6%, 45.1%	Improve semen parameters	1. Absence of a placebo control group 2. Geographical limitations of single-center studies 3. Potential bias due to the single-blind study design	Ma et al. (2022) (137)
Composite formula	L-carnitine + CoQ10	Randomized controlled trial	262	10 ml of L-carnitine solution orally twice a day and 20 mg of CoQ10 tablets orally three times a day, 3 months	The sperm concentration and progressive motility increased by 16.2%, 40.8%, DNA fragmentation decreased by 25.3%	Significantly improve semen parameters and pregnancy outcomes	1. Absence of a placebo control group 2. Geographical limitations of single-center studies	Cheng et al. (2018) (122)
	Multinutrient complex	Randomized controlled trial	50	30 mg of CoQ10, 8 mg of zinc, 100 mg of vitamin C, 12 mg of vitamin E, 400 mg of folic acid once a day as well as 200 mg of selenium every other day, 3 months	The sperm cell concentration and sperm motility increased by 73.4%, 46.4%	The synergistic effect of multiple mechanisms	1. Potential bias due to the single-blind study design 2. Absence of a placebo control group 3. Geographical limitations of single-center studies	Sadaghiani et al. (2020) (123)
New formulation	Lycopene	Randomized controlled trial	44	25 mg/day, 3 months	Ejaculate volume, sperm concentration, total count and total motility increased by 20.2%, 64.3%, 107.1%, 49.2%	Plant antioxidants have potential applications	Geographical limitations of single-center studies	Nouri et al. (2019) (138)

Despite existing controversies, antioxidant intervention remains one of the primary treatments for OS-related male infertility (124). However, it should be noted that excessive supplementation may induce “reductive stress”, which can adversely affect cellular function (58). Therefore, clinically it is recommended to use moderate-dose, combined antioxidant regimens—such as vitamins C and E along with other small-molecule antioxidants—to balance efficacy and safety.

Beyond simple antioxidant supplementation, comprehensive management strategies show significant potential. Measures such as reducing exposure to environmental toxins (e.g., heavy metals and pesticides), improving lifestyle habits (e.g., smoking cessation and alcohol moderation), and increasing the intake of natural antioxidants (e.g., fruits, vegetables, and nuts) all contribute to alleviating OS and improving mitochondrial function (125–127). Novel regulatory approaches, including resveratrol, flavonoids, and mitochondria-targeted agents, may further enhance sperm quality by improving mitochondrial membrane potential and energy metabolism (128–132).

In summary, antioxidant therapy holds promise in the management of asthenozoospermia, though current understanding of its dose-response relationships and mechanisms remains incomplete. Future research should focus on clarifying the efficacy and mechanisms of specific antioxidants and dosages, as well as exploring integrated treatment strategies that combine antioxidants with other approaches—such as lifestyle modifications, pharmacological agents, and assisted reproductive technologies—to provide new avenues for improving male infertility.

## 5 Conclusions

Male infertility is a significant global health issue affecting couples of reproductive age, with asthenozoospermia being one of its major clinical manifestations. This review systematically examines the central role of OS in the pathogenesis of asthenozoospermia. OS arises from an imbalance between oxidative and antioxidant systems. Excessive ROS attack polyunsaturated fatty acids in the sperm membrane, triggering lipid peroxidation and compromising membrane integrity. Meanwhile, ROS induce nuclear DNA fragmentation, impairing genetic stability. Crucially, as the primary site of ROS generation and cellular energy production, mitochondria suffer structural and functional impairments under OS, directly leading to reduced sperm motility and activation of autophagic pathways. Based on these mechanisms, current research focuses on lifestyle modifications, nutritional interventions, and antioxidant therapies to alleviate OS, improve mitochondrial function, and ultimately improve sperm quality.

Nevertheless, this field still faces critical challenges. Antioxidant therapy acts as a “double-edged sword” due to the dual role of ROS as signaling molecules and damaging agents, resulting in heterogeneous treatment outcomes. A transition toward personalized medicine is therefore essential, requiring precise patient stratification, optimized dosing regimens, and reliable biomarkers of OS. At the same time, a deeper understanding of mitochondrial multifunctionality—spanning energy metabolism, signaling, and apoptosis—is imperative. Therapeutic strategies must evolve beyond energy support to target mitochondrial dynamics, mtDNA integrity, and mitophagy. Rigorous evaluation of the safety and specificity of mitochondrial-targeted compounds remains necessary. In summary, OS and mitochondrial dysfunction are central to asthenozoospermia pathogenesis. Future research should prioritize defining physiological-pathological ROS thresholds, establishing standardized diagnostic frameworks, validating personalized interventions, and prospectively assessing novel therapeutics. Mechanism-driven interdisciplinary collaboration will be key to advancing precision medicine in this field.
